# Transcriptome analysis of *Cymbidium sinense* and its application to the identification of genes associated with floral development

**DOI:** 10.1186/1471-2164-14-279

**Published:** 2013-04-24

**Authors:** Jianxia Zhang, Kunlin Wu, Songjun Zeng, Jaime A Teixeira da Silva, Xiaolan Zhao, Chang-En Tian, Haoqiang Xia, Jun Duan

**Affiliations:** 1 Key Laboratory of South China Agricultural Plant Genetics and Breeding, South China Botanical Garden, The Chinese Academy of Sciences, Guangzhou, 510650, China; 2 Key Laboratory of Plant Resources Conservation and Sustainable Utilization, South China Botanical Garden, Chinese Academy of Sciences, Guangzhou, 510650, China; 3 Faculty of Agriculture and Graduate School of Agriculture, Kagawa University, Miki-cho, Kagawa, 761-0795, Japan; 4 Guangdong Key Laboratory for Innovative Development and Utilization of Forest Plant Germplasm, South China Agricultural University, Guangzhou, 510642, China; 5 School of Life Sciences, Guangzhou University, Guangzhou, 510006, China; 6 Guangzhou Genedenovo Biotechnology Co.,Ltd, Guangzhou, 510006, China

**Keywords:** Floral development, Flowering time, Digital gene expression, Transcriptome, *Cymbidium sinense*

## Abstract

**Background:**

*Cymbidium sinense* belongs to the Orchidaceae, which is one of the most abundant angiosperm families. *C. sinense*, a high-grade traditional potted flower, is most prevalent in China and some Southeast Asian countries. The control of flowering time is a major bottleneck in the industrialized development of *C. sinense*. Little is known about the mechanisms responsible for floral development in this orchid. Moreover, genome references for entire transcriptome sequences do not currently exist for *C. sinense*. Thus, transcriptome and expression profiling data for this species are needed as an important resource to identify genes and to better understand the biological mechanisms of floral development in *C. sinense*.

**Results:**

In this study, *de novo* transcriptome assembly and gene expression analysis using Illumina sequencing technology were performed. Transcriptome analysis assembles gene-related information related to vegetative and reproductive growth of *C. sinense.* Illumina sequencing generated 54,248,006 high quality reads that were assembled into 83,580 unigenes with an average sequence length of 612 base pairs, including 13,315 clusters and 70,265 singletons. A total of 41,687 (49.88%) unique sequences were annotated, 23,092 of which were assigned to specific metabolic pathways by the Kyoto Encyclopedia of Genes and Genomes (KEGG). Gene Ontology (GO) analysis of the annotated unigenes revealed that the majority of sequenced genes were associated with metabolic and cellular processes, cell and cell parts, catalytic activity and binding. Furthermore, 120 flowering-associated unigenes, 73 *MADS-box* unigenes and 28 *CONSTANS-LIKE* (*COL*) unigenes were identified from our collection. In addition, three digital gene expression (DGE) libraries were constructed for the vegetative phase (VP), floral differentiation phase (FDP) and reproductive phase (RP). The specific expression of many genes in the three development phases was also identified. 32 genes among three sub-libraries with high differential expression were selected as candidates connected with flower development.

**Conclusion:**

RNA-seq and DGE profiling data provided comprehensive gene expression information at the transcriptional level that could facilitate our understanding of the molecular mechanisms of floral development at three development phases of *C. sinense.* This data could be used as an important resource for investigating the genetics of the flowering pathway and various biological mechanisms in this orchid.

## Background

The Orchidaceae is one of the largest and most widespread families of flowering plants, with more than 250000 species [1]. *Cymbidium*, a very important economically flowering genus with the Orchidaceae, has nearly 55 species throughout the world and is widespread in tropical and subtropical Asia, reaching as far south as Papua New Guinea and Australia [2]. *Cymbidium sinense* is a perennial terrestrial species native to China and has more than a thousand years of cultural history. It holds a strong position in traditional flower markets in China, Japan, Korea, and Southeast Asia. It is of great horticultural value as an ornamental plant because of its elegance, its upright leaves, and beautiful and fragrant flowers. *C. sinense* blooms in winter from January to March and is usually regarded as a Spring Festival flower. Although *C. sinense* is a valuable orchid, its flowering pathway is not clearly understood. Variation in environmental conditions, including light, temperature and hormones, is likely to regulate flowering time and flower quality. Flowering regulation technology for *C. sinense* is currently scarce on the market, which seriously hinders the development of the orchid industry and also reduces the economic value of *C. sinense.*

The transition from vegetative growth to flowering is very importance because flowering is the first step of sexual reproduction [3]. In *Arabidopsis thaliana*, flower initiation takes place via four (gibberellin, autonomous, vernalization, light-dependent) genetic pathways. These processes are integrated by the function of the *FLOWERING LOCUS D* (*FD*), *FLOWERING LOCUS E (FE)*, *FLOWERING WAGENINGEN* (*FWA*), *PROTODERMAL FACTOR2* (*PDF2*), *SUPPRESSOR OF OVEREXPRESSION OF CO 1*(*SOC1*), and *FLOWERING LOCUS T* (*FT*) genes. The integrated signal of floral induction is transmitted to the floral meristem identity genes *LEATY* (*LFY*) and *APETALA1* (*AP1*), after which floral morphogenesis takes place [4]. Currently only a few flowering genes have been cloned from orchids. *DOH1* and *DOMADS1* were isolated and identified from *Dendrobium* Madame*. DOH1* plays a negative regulatory role in floral formation while *DOMADS1* is a marker gene specifically expressed in the shoot apical meristem during floral transition. *DOH1* is a possible upstream regulator of *DOMADS1* and inhibits its expression [5,6]. Some *MADS-box* function genes were also isolated from *Dendrobium crumenatum.* They include *APETALA2 (AP2)*, *PISTILLATA (PI)/GLOBOSA (GLO)*, *APETALA3* (*AP3*) */DEFIENCE (DEF)-*like, *AGAMOUS (AG)* and *SEPALLATA (SEP)* genes, which play an important role in the early regulation of the conversion to flowers [7]. *OMADS3* was isolated and characterized from *Oncidium* Gower Ramsey with a function similar to a functional gene regulating flower formation as well as floral initiation [8]. *OMADS1* from *O.* Gower Ramsey promoted flower transition and formation by acting as an activator for *FT* and *SOC1* in *Arabidopsis. OMADS1* was able to strongly interact with *OMADS3*, which influenced flower formation and floral initiation [9].

Compared with other orchids, very little research exists on the role of flowering genes in the regulation of the vegetative-to-flowering transition and flower initiation in *Cymbidium*. Few reports investigating the functions of flowering time genes of *Cymbidium* exist. Genomic resources available for the species are also scarce. Together with the nucleotide sequences obtained by NCBI searches, 60 *Cymbidium* expressed sequence tags (ESTs) are, however, available. Nevertheless, this genetic data is insufficient for elucidating the molecular mechanism of floral regulation in *C. sinense*.

In recent years, Illumina sequencing techniques have provided fascinating opportunities in life sciences and dramatically improved the efficiency of gene discovery. However, the entire transcriptome of *C. sinense* has not been sequenced. In this study, RNA-seq and digital gene expression (DGE) were performed using Illumina technology. Illumina sequencing provided comprehensive information about gene expression at the transcriptional level that could facilitate our understanding of the molecular mechanisms of *C. sinense* floral development*.* Such data for *C. sinense* could also be used as an important resource to investigate the flowering pathway and various other biological mechanisms in other orchid species.

## Results

### Illumina sequencing and sequence assembly

**Figure 1:**
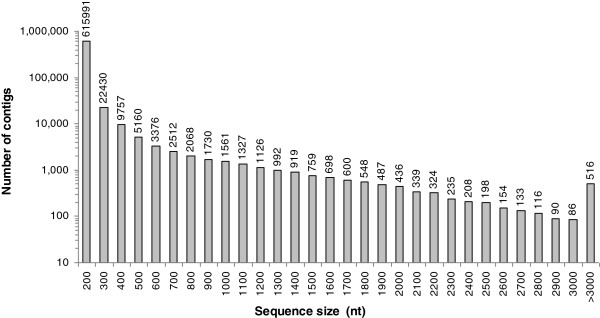
**The length distribution of assembled *C. sinense *contigs.**

**Figure 2:**
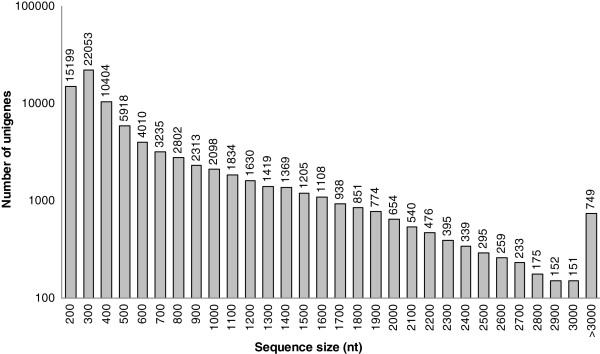
**The length distribution of assembled *C. sinense *unigenes.**

To obtain an overview of the *C. sinense* transcriptome, a cDNA library was generated from an equal mixture of RNA isolated from all organs, and pair end sequenced using the Illumina Hiseq™2000 platform. After cleaning and quality checks, 54 million 90-bp reads were assembled into 147162 contigs with a mean length of 326 bp (Table 1). Using paired-end reads, these contigs were further assembled into 83580 unigenes by Trinity, including 13315 clusters and 70265 singletons, with a mean length of 612 bp. The size distribution of these contigs and unigenes are shown in Figures 1 and 2. The assembly produced a substantial number of large contigs: 11852 contigs were >1,000 bp in length and 26698 contigs were >500 bp, although most contigs were between 200 and 300 bp in length (Figure 1). 17644 unigenes were >1,000 bp in length (Figure 2).

**Table 1:** **Summary for *C. sinense *transcriptome**

Total number of raw reads	59,512,598
Total number of clean reads	54,248,006
Total clean nucleotides (nt)	4,882,320,540
Average read length	90
Total number of contigs	147,162
Mean length of contigs	326
Total number of unigenes	83,580
Mean length of unigenes	612

### Annotation of predicted proteins

After searching the reference sequences using BLASTX against nr, SwissPort, COG and KEGG, we found a total of 41687 (49.88% of all unigenes) unigenes providing a significant BLAST result (Table 2).

**Table 2:** **Statistics of annotation results for *C. sinense *unigenes**

**Sequence file**	**All**	**NR**	**SwissPort**	**KEGG**	**COG**	**GO**
JX-Unigene	41,687	41,161	30,606	23,092	15,041	16,565

**Figure 3:**
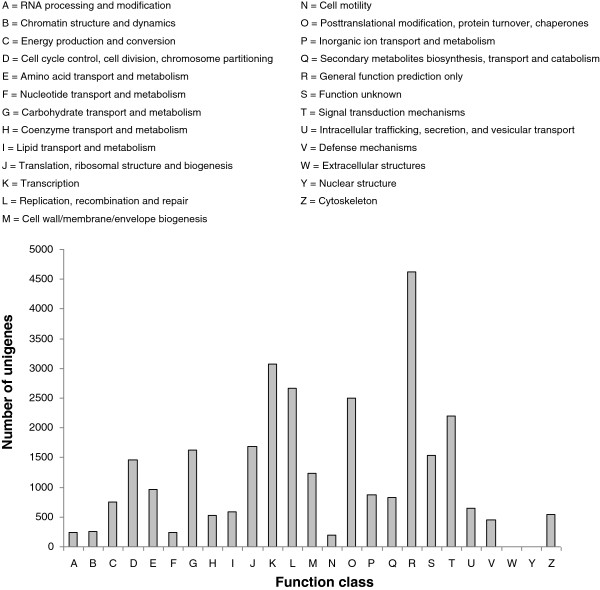
**COG function classification of *C. sinense *unigenes.**

Among the 41687 unigenes, approximately 36.1% could be annotated in COG based on sequence homologies. In the COG classification, 15041 unigenes were classified into 25 function classifications (Figure 3). ‘General function prediction’ was dominant. ‘Translation’, ‘replication, recombination and repair’ and ‘posttranslational’ also shared a high-percentage of genes among the categories, and only a few genes matched the terms ‘nuclear structure’ and ‘extracellular structures’. 2199 unigenes were annotated as the ‘signal transduction mechanisms’ category, which suggests that our study may allow for the identification of novel genes involved in signal transduction pathways. The COG analysis showed that the identified genes are involved in various biological processes.

### GO classification for unigenes

**Figure 4:**
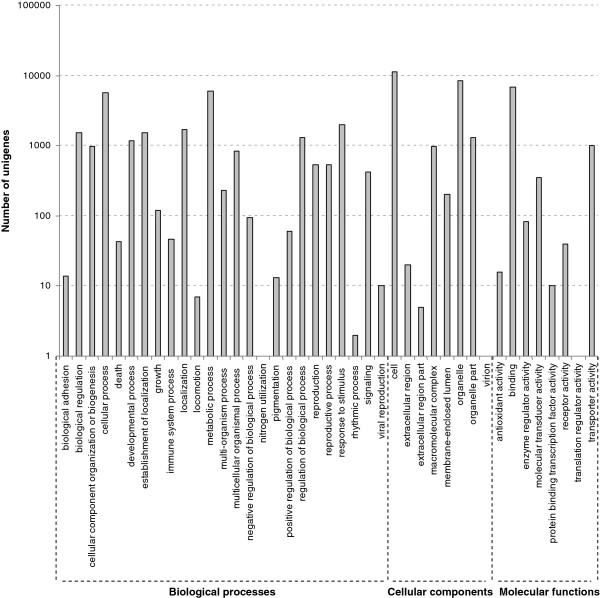
**GO classification of *C. sinense *unigenes.**

We used GO assignments to classify the functions of the predicted *C. sinense* unigenes. 16565 annotated unigenes were further categorized into 44 functional groups (Figure 4). Metabolic and cellular processes were the most highly represented groups in the biological process category. 5986 unigenes were annotated as the ‘metabolic process’ category, which suggests that our study may allow for the identification of novel genes involved in secondary metabolite synthesis pathways. Cell and cell parts were dominant groups in the “cellular component function” category. In the “molecular function” category, a high percentage of genes came from the ‘binding’ (41.1%) and ‘catalytic activity’ (44.6%) groups. In the ‘nitrogen utilization’, ‘virion’ and ‘translation regulator activity’ groups, only a single unigene was annotated for each (Figure 4).

### Metabolic pathway assignment by KEGG

In our study, 41687 annotated sequences were mapped to the reference canonical pathways in KEGG. In total, 23092 sequences were assigned to 126 KEGG pathways (Additional file 1). The pathways with most representation by the unigenes were metabolic pathways (5179 members, 22.43%) and biosynthesis of secondary metabolites (2161 members, 9.36%). These pathways provide a valuable resource for investigating specific processes, functions and pathways during *C. sinense* research. Interestingly, 1397 unigenes involved in plant hormone signal transduction were found, which contained 9 pathways (Table 3) controlling the signal transduction of several plant growth regulators, for example, auxin, cytokinin, gibberellin, abscisic acid, ethylene, brassinosteroid, jasmonic acid and salicylic acid. These hormones regulate seed dormancy, plant vegetative and reproductive growth, fruit ripening senescence and stress response.

**Table 3:** The pathways and the products involved in the pathway of plant hormone signal transduction

**Pathway**	**Product**	**Pathway ID**
Brassinosteroid biosynthesis	Brassinosteroid	ko00905
Carotenoid biosynthesis	Abscisic acid	Ko00906
Cysteine and methionine metabolism	Ethylene	Ko00270
Diterpenoid biosynthesis	Gibberellin	Ko00904
Indole alkaloid biosynthesis	Indole-acetic acid	Ko00901
α-Linolenic acid metabolism	Jasmonic acid	Ko00591
Phenylalanine metabolism	Salicylic acid	Ko00360
Tryptophan metabolism	Auxin	Ko00380
Zeatin biosynthesis	Cytokinin	ko00908

**Figure 5:**
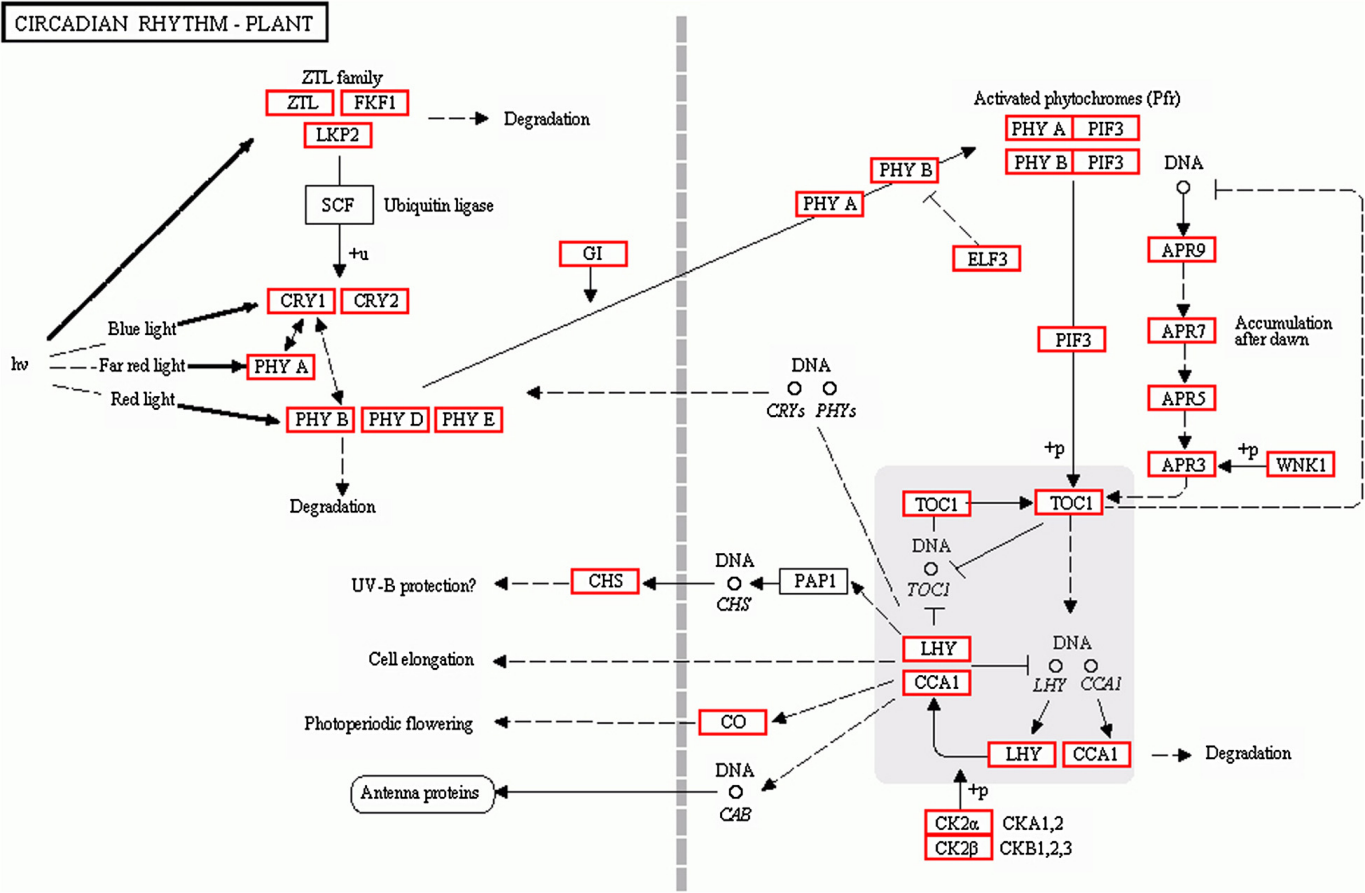
Metabolic pathway of the circadian rhythm for unigenes by KEGG annotation.

The circadian clock is an important factor controlling plant physiology, and is also an important part of the photoperiod pathway. It regulates physiological activities by controlling the circadian rhythm [10]. A circadian rhythm was found in the KEGG pathway involving 222 (0.97%) unigenes. The detailed metabolic pathway for the circadian rhythm is shown in Figure 5. Every gene in the pathway was associated with several unigenes. The pathway will be useful for further studies on the effect of the photoperiod pathway on *C. sinense* flowering-related processes.

### Identifying *C. sinense* flowering time-associated genes and MADS-box genes

According to the annotation of unigenes, we obtained 120 genes associated with flowering time. Some genes are shown in Table 4. These include photoperiod pathway genes such as *GIGANTEA (GI)*, *EARLY FLOWERING 3* (*EIF3)*, *PHYTOCHROME INTERACTING FACTOR 3* (*PIF3)*, *LATE ELONGATED HYPOCOTYL (LHY)*, *CHALCONE SYNTHASE (CHS)*, *CIRCADIAN CLOCK ASSOCIATED 1* (*CCA1)*, and *CO*; vernalization pathway genes related to *VERNALIZATION (VRN)*; floral integrator pathway genes related to *CAULOFLOWER (CAL)*, *AP2*, *FT*, *SOC1* and *TERMINAL FLOWER 1(TFL)*; and floral meristem identity genes *LFY* and *AP1* were all identified in our *C. sinense* transcriptome database. Additionally, 73 MADS-box unigenes were also discovered (Additional file 2). All these unigenes are important resources for the study of floral development and flower organ formation in the future.

**Table 4:** ***C. sinense*unigenes that share homology with flowering time genes**

**Category**	**Gene ID**	**Homologous gene**	**Nr-ID**
Photoperiod pathway	CL636.Contig1_JX	GI	ADP92454.1
	CL8490.Contig1_JX		ADP92454.1
	Unigene61105_JX	ELF3	ABL11477.1
	Unigene61030_JX	PIF3	NP_001063455.1
	Unigene31120_JX	LHY	ACF60466.1
	Unigene14045_JX	CHS	NP_001064831.1
	CL2407.Contig1_JX		O23729.1
	Unigene68739_JX	CCA1	ABW87009.1
	CL1617.Contig2_JX	CO	NP_001047975.1
	CL1617.Contig1_JX		NP_001057441.1
	CL10349.Contig1_JX		NP_001062363.2
	CL10838.Contig1_JX		ADL36678.1
Vernalization pathway	Unigene49191_JX	VRN2	ABD85301.1
	Unigene49192_JX		ABD85301.1
	Unigene50102_JX		ABD85301.1
Floral integrator pathway	Unigene4182_JX	FT/HD3a	ADI58462.1
	Unigene43540_JX		ABX11019.1
	Unigene7817_JX		CBY25182.1
	CL743.Contig1_JX		ADP89470.1
	CL743.Contig2_JX		ADP89470.1
	Unigene35813_JX	TFL	ACX53295.1
	Unigene36169_JX		BAD02372.1
	CL11326.Contig1_JX	AP2	ABB90554.1
	CL12341.Contig1_JX		NP_001147356.1
	CL1488.Contig1_JX		AAZ95247.1
	CL1700.Contig1_JX		AAZ66389.1
	CL2122.Contig1_JX		XP_002304138.1
	CL260.Contig1_JX		XP_002274890.1
	CL260.Contig2_JX		XP_002274890.1
	CL260.Contig3_JX		XP_002274890.1
	CL260.Contig4_JX		XP_002274890.1
	CL3589.Contig1_JX		XP_002325111.1
	Unigene32766_JX	SOC1	ADP06386.1
	Unigene59851_JX		ADJ67241.1
	Unigene32196_JX		BAC55080.1
	Unigene10614_JX		ADJ67238.1
	Unigene59851_JX	CAL	ADJ67241.1
	Unigene10812_JX		ADI58464.1
Floral meristem identity	Unigene32196_JX	LFY	BAC55080.1
	Unigene630_JX		AAY40170.1
	Unigene2289_JX	AP1	ADP00515.1
	CL162.Contig1_JX		ADJ67240.1
	CL162.Contig2_JX		ADJ67240.1

### Identifying *C. sinense CONSTANS*-like gene family

*CO* plays a central regulatory role in the photoperiod pathway, and is regulated by the circadian clock and light [11,12]. To determine the *COL* gene family of *C. sinense*, we analyzed the transcriptome database generated by this study and found 28 unigenes annotated as zinc finger protein CONSTANS. As shown in Additional file 3, they showed homology with 12 *Arabidopsis COL* genes.

**Figure 6:**
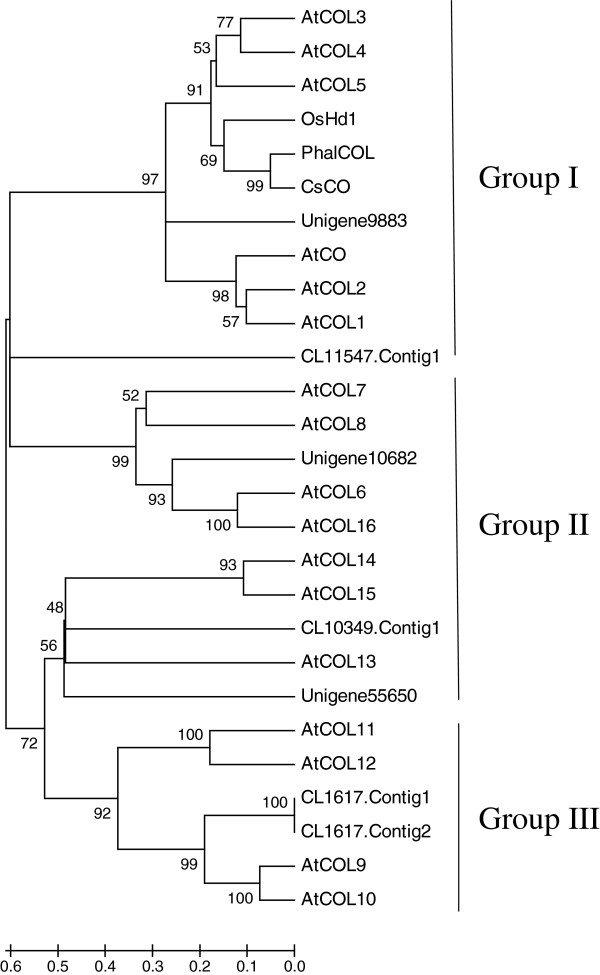
**Phylogenetic analysis of the CONSTANS or CONSTANS-like proteins from different plant species.** The tree is displayed as a phylogram in which branch lengths are proportional to distance. Bootstrap values for 1,000 replicates were used to assess the robustness of the trees. The proteins are as follows: AtCO (accession no. NP_197088), AtCOL1 (accession no. NP_197089), AtCOL2 (accession no. NP_186887), AtCOL3 (accession no. Q9SK53), AtCOL4 (accession no. Q940T9.2), AtCOL5 (accession no. Q9FHH8), AtCOL6 (accession no. Q8LG76), AtCOL7 (accession no. Q9C9A9), AtCOL8 (accession no. Q9M9B3), AtCOL9 (accession no. NP_001118599), AtCOL10 (accession no. Q9LUA9), AtCOL11 (accession no. O23379), AtCOL12 (accession no. Q9LJ44), AtCOL13 (accession no. O82256), AtCOL14 (accession no. O22800), AtCOL15 (accession no. Q9C7E8), AtCOL16 (accession no. Q8RWD0), OsHd1 (accession no. ABB17664), PhalCOL (accession no. FJ469986), CsCO (accession no. GU168786).

We selected seven unigenes which have two conserved B-box domains and a CCT domain to further examine their relationship with other *COL* genes from *Arabidopsis*, rice, *Phalaenopsis* and *C. sinense.* The seven unigenes are shown in Table 5. Their amino acid sequences were used to construct a phylogenetic tree using MEGA4 [13] to determine genetic distances. The phylogenetic tree was showed that all members could be divided into three divergent groups (Figure 6). Unigene9883 and CL11547.Contig1 were clustered with *CsCO*, *PhalCOL* as well as *OsHd1* in group I. They are closely related to *Arabidopsis AtCO/AtCOL1/AtCOL2*. CL10349.Contig1, Unigene55660, CL1617.Contig1 and CL1617.Contig2 were clustered in group II. CL1617.Contig1 and CL1617.Contig2 are closely related to *AtCOL9/AtCOL10*. Unigene55650 is closely related to *AtCOL13*, and CL10349.Contig1 is closely related to *AtCOL14/AtCOL15*. Unigene10682 was assigned to group III and is closely related to *AtCOL6/AtCOL16*. These results are consistent with the data shown in Table 5.

**Table 5:** ***C. sinense *unigenes that share homology with *CONSTANS-like *genes of *Arabidopsis***

**Gene ID**	**Length of gene (bp)**	**Swissport annotation**	**Swissport-ID**
CL11547.Contig1_JX	955	*Arabidopsis thaliana* COL1	O50055
Unigene9883_JX	1023	*Arabidopsis thaliana* COL2	Q96502
Unigene10682_JX	1636	*Arabidopsis thaliana* COL6	Q8LG76
CL1617.Contig1_JX	1957	*Arabidopsis thaliana* COL9	Q9SSE5
CL1617.Contig2_JX	2043	*Arabidopsis thaliana* COL9	Q9SSE5
Unigene55650_JX	1392	*Arabidopsis thaliana* COL13	O82256
CL10349.Contig1_JX	784	*Arabidopsis thaliana* COL14	O22800

### DGE library sequencing and evaluation

The materials from VP, FDP, and RP were chosen to construct three DGE libraries, then which were sequenced. Three DGE sequencing quality evaluation and alignment statistics were shown in Table 6. The percent of all low quality reads including only adaptor and containing N reads was less than 0.5% in three libraries. 99.5% of raw tags in each library were clean tags. After filtering the low quality tags, the total number of clean tags in each library was 11.98, 12.65 and 12.16 million, respectively. Among the clean tags, the number of sequences that could be mapped to transcriptome sequences amounted to 10.79, 11.85 and 10.71 million, corresponding to 90.10, 93.67 and 88.07% in the three libraries, respectively.

**Table 6:** DGE sequencing quality evaluation and alignment statistics

**Summary**	**VP**	**FDP**	**RP**
Only adaptor reads	25804	19835	37799
Only adaptor reads%	0.21%	0.16%	0.31%
Containing N reads	37	40	43
Containing N reads%	0.00%	0.00%	0.00%
Low quality reads	25686	32107	16577
Low quality reads%	0.21%	0.25%	0.14%
Total clean reads	11981512	12647899	12164959
Total clean reads%	99.57%	99.59%	99.55%
Total base pairs	587094088	619747051	596082991
Total mapped reads to gene	10795616	11847282	10713910
Total% of mapped reads	90.10%	93.67%	88.07%
Perfect match	7558927	10028451	8649669
Perfect match%	63.09%	79.29%	71.10%
≤2 bp mismatch	3236689	1818831	2064241
≤2 bp mismatch%	27.01%	14.38%	16.97%
Unique match	9389031	10763499	8207609
Unique match%	78.36%	85.10%	67.47%
Multi-position match	1406585	1083783	2506301
Multi-position match%	11.74%	8.57%	20.60%
Total unmapped reads	1185896	800617	1451049
Total unmapped reads%	9.90%	6.33%	11.93%

### Variation in gene expression among three developmental stages

To identify the differentially expressed genes during three developmental stages, the number of clean tags for each gene was calculated, and the genes that were differentially expressed between the two samples were identified according to the method described by Audic and Claverie [14].

**Figure 7:**
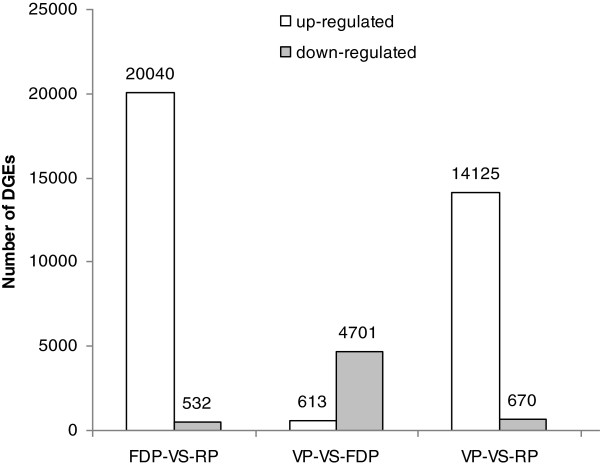
Numbers of DGE unigenes in each comparison.

The variations in gene expression were analyzed by comparing VP and FDP, VP and RP, and FDP and RP. The comparison between VP and FDP revealed significant variations in expression. A total of 5314 genes, including 613 up-regulated and 4701 down-regulated genes, were identified in FDP compared to VP (Figure 7). 34 genes showed specific expression in FDP and 107 genes showed specific expression in VP. These specific expression genes were listed in Additional file 4.

Based on the GO functional classification, most of the differential expression gene sets demonstrated down-regulated expression in the FDP library, and these genes were mainly correlated to membrane, vesicle and cellular component organization (Additional file 5). In the KEGG classification, 6 gene sets were significantly enriched, and most of these genes were mainly down-regulated in the FDP library and correlated to plant hormone signal transduction (Additional file 5).

Meanwhile, we compared the variations in gene expression between VP and RP. A total of 14795 genes, including 14125 up-regulated and 761 down-regulated genes, were identified in RP compared to VP (Figure 7). 1408 genes showed specific expression in RP and 38 genes showed specific expression in VP. Those genes that showed specific expression were listed in Additional file 6.

Based on the GO functional classification, most of the gene sets demonstrated up-regulated expression in the RP library, and these genes were mainly correlated to hydrolase activity, binding and organelle parts (Additional file 7). In the KEGG classification, 13 gene sets were significantly enriched, and most of these genes were mainly up-regulated in the FDP library and correlated to the spliceosome and ribosome (Additional file 7).

A comparison between FDP and RP revealed more variations in gene expression. The results revealed 20572 genes with significant differential expression levels between FDP and RP. Among them, 20040 and 532 genes were up-regulated and down-regulated, respectively (Figure 7). 2535 genes showed specific expression in RP and 25 genes showed specific expression in FDP. Those genes that showed specific expression are listed in Additional file 8.

Based on the GO functional classification, most of the gene sets demonstrated up-regulated expression in the RP library, and these genes were mainly correlated to binding, membrane-bounded organelles and organelle parts (Additional file 9). In the KEGG classification, 12 gene sets were significantly enriched, and most of these genes were mainly up-regulated in the RP library and correlated to the spliceosome and RNA transport (Additional file 9).

**Figure 8:**
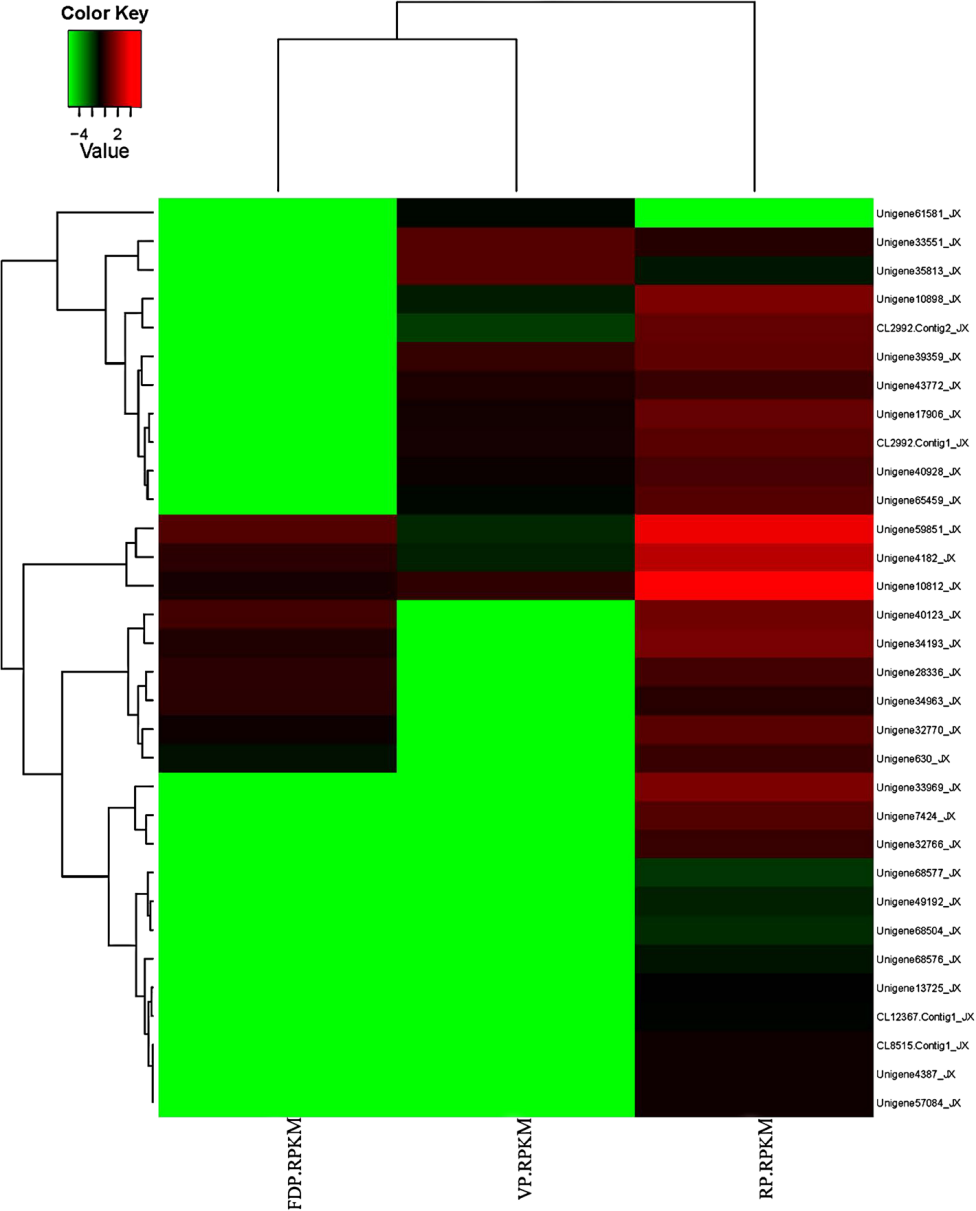
**Differential expression genes related with flower development in *C. sinense *DGE.** Each column represents an experimental sample (e.g. VP, FDP and RP) and each row represents a gene. Expression differences are shown in different colors. Red means high expression and green means low expression.

**Figure 9:**
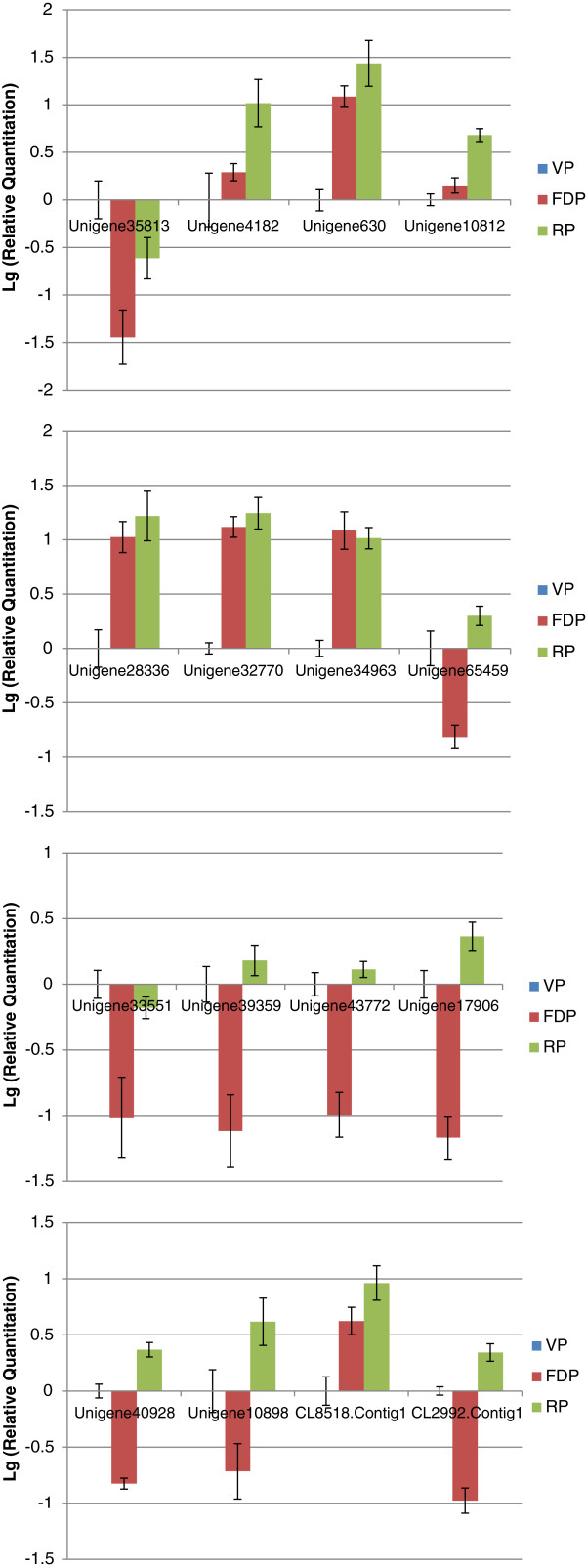
**The qRT-PCR analysis of gene expression in VP (blue bars), FDP (red bars) and RP (green bars).** The y-axis indicates fold change in expression among the samples VP, FDP, and RP using the results from RT-qPCR. The Lg(Relative Quantitation) of 16 genes in the VP was calibrated as zero.

We also focused on the genes related to flower development and chose some genes that showed significantly different expression among three developmental phases (Figure 8). The functional annotation for these unigenes was listed in Additional file 10. For example, Unigene40123 demonstrated higher expression levels in FDP and RP than in VP, Unigene33969 showed higher expression levels in RP than in VP and FDP, and Unigene65459 showed higher expression levels in VP and RP than in FDP, which were all homologous with *Arabidopsis thaliana* auxin response factor 2 (ARF2) and related to auxin metabolism. Other significant expression of different genes among the three developmental phases might be associated with circadian rhythm (Unigene4387, 33551, 35813, homolog with *FD*), the vernalization pathway (Unigene49193 and CL2992.Contig2, homologous with *EMBRYONIC FLOWER2 (EMF2)* and *VERNALIZATION INSENSITIVE 3 (VIN3)*, respectively and so on. To validate DGE-tag profiling, 16 genes were selected randomly to examine the expression using RT-qPCR (Figure 9). The results were exhibited differential expression among three libraries and were identical to those obtained by DGE expression profiling. Thus, the data generated in this study is sufficient to be used as a tool to investigate some specific flowering genes which show comparative expression levels among different developmental phases.

## Discussion

### Illumina sequencing and sequence annotation

*C. sinense* is a very popular and traditional orchid in China. However, little is known about the mechanisms responsible for floral development and genomic information for *C. sinense* is currently unavailable. The aims of this project were to generate a large amount of cDNA sequence data that would facilitate more detailed studies in *C. sinense*, and to identify the genes controlling flowering time. The availability of transcriptome data for *C. sinense* will meet the initial information needs for functional studies of this species and its relatives. In this study, a combination of RNA-seq and three DGE analyses were performed using Illumina sequencing, which generated 54,248,006 high quality reads that were assembled into 83580 unigenes with an average sequence length of 612 base pairs. These unigenes were used for BLASTX and annotation against protein databases like nr, SwissPort, COG, KEGG and GO. In total, 41687 sequences were identified through BLAST searches and 50.1% unigenes had no homologues in the NCBI database. This may indicate that *C. sinense* vegetative and reproductive growth contains many unique processes and pathways.

### Identifying *C. sinense* flowering time-associated genes

The photoperiod and vernalization pathways are two important genetic networks of flowering control [4]. In our study, the sequences associated with both pathways could be identified (Table 4). The photoperiod pathway comprises three parts: photoreceptors, a circadian clock and an output pathway from the clock specific to flowering [15]. Light signals are first received by two photoreceptors, phytochromes (PhyA, PhyB, PhyC, PhyD, PhyE) and cryptochromes (Cryl, Cry2) [16-20], which process the physical signals and produce a circadian clock [21-23]. The circadian clock provides a seasonal signal that adjusts plant development and regulates flowering time. It is controlled by a group of gene complexes that are composed of *TOC1*, *CCA1* and *LHY* and regulate transcriptional–translational negative feedback loops [24-26]. *ELF3* affects light input to the oscillator [27,28]. The processed signal is transmitted to the *GI* gene and the resultant signal activates the *CO* gene [11]. *GI* is another clock-associated protein known to regulate circadian oscillation and flowering time of *Arabidopsis*[29,30]. The *gi* mutants are defective for the expression of *CCA1* and *LHY* genes. The circadian clock controls the expression rhythm of *CO* through *GI*[31]. *CO* is a transcription factor that promotes flowering by inducing the expression of the direct downstream genes *FT*[32] and *SOC1*[33]. *COL* genes have been identified in some plant species, each of which seems to have a large family of these genes [34]. In *Arabidopsis*, there are 17 *COL* genes [35]. There are at least 16 *COL* genes in the rice genome [34]. These COL proteins contain two B-box domains at the N-terminus and a CCT domain at the C-terminus [35,36]. In this study, 28 *C. sinense COL* unigenes containing the conserved CCT domain were identified (Additional file 3). Seven unigenes were used to perform the phylogenetic analysis. They were classified into three conserved *COL* subgroups as defined previously [35]. COL proteins belonging to different subgroups are expected to perform distinct biological roles, but only several *COL* genes controlling flowering time have been studied in detail [34]. We presume that seven unigenes in *C. sinense* are expected to perform distinct biological roles. Sequence homologues for other genes involved in regulation of the circadian clock described above (*GI*, *EIF3*, *PIF3*, *LHY*, *CHS*, *CCA1*) could be found in our database (Table 4). Furthermore, detailed metabolic pathways for the circadian rhythm and 222 related unigenes, such as phytochromes (PhyA, PhyB, PhyC, PhyD, PhyE) and cryptochromes (Cryl, Cry2) were found (Figure 5). Thus, these photoperiod pathways may be concerned with the regulation of flowering time in *C. sinense.*

*VRN2* is a key gene involved in the vernalization pathway [37]. It promotes flowering by repressing the expression of *FLOWERING LOCUS C (FLC)*[38]. *VRN2* codes for a protein with homology to PcG proteins [39]. Thus, *VRN2* may function to keep the *FLC*-chromatin state for down-regulation. Sequence homologs for *VRN2* could be found in our database (Table 4). Furthermore, temperature obviously affects the flowering time of *C. sinense*. We speculate that the vernalization pathway is related to the regulation of flowering time in *C. sinense*.

### Identifying *C. sinense* flowering integration genes and floral meristem identity genes

Flowering integration genes accept the signal from the genetic pathway, and then induce floral meristem identity (FMI) genes for flowering as a whole [4]. At present, three integration genes, including *SOC1/AGL20*, *FT* and *TFL*, have been identified. *SOC1* and *FT* are the direct target genes for *CO*. The expression of *FT* and *SOC1* is controlled positively not only by the light pathway, but also by the autonomous pathway acting negatively through *FLC*[40]. The vernalization signal increases *SOC1* expression, presumably by reducing *FLC* levels, and *SOC1* can also be up-regulated by a gibberellin pathway. Accordingly, *SOC1* and *FT* act as the convergence of all four pathways [41]. *TFL1* codes for a protein with homology to *FT*[42]. The mutations in *TFL1* are semidominant and early flowering with a determinate inflorescence [43]. Thus, *TFL1* codes for a repressor of flowering. *TFL2* functions as a negative repressor of *FT* expression. Sequence homologs for the floral integrator pathway genes related to *FT*, *SOC1* and *TFL* were all identified in our *C. sinense* transcriptome database (Table 4).

The floral meristem is initiated by a set of FMI genes that include *LFY*, *AP1*, *CAL*, *AP2*, and *UNUSUAL FLORAL ORGANS (UFO)*[44-47]. Among them, *LFY* and *AP1* play a central role in flower meristem identity [48-50]. *LFY* regulates inflorescence shape and controls flowering time [51,52]. *AP1* is the downriver target gene of *LFY. LFY* induces expression of *AP1*. LFY protein, combined with the *AP1* promoter activates the transcription of *AP1*[53]. The phenotype of the loss-of-function-type mutations in *LFY* or *AP1* gene is as follows: Flowers either have vegetative characteristics or have been replaced by vegetative shoots [54]. The phenotype of double mutations in *lfy/ap1* is a scarce floral structure [49,55], which elucidated the functional redundancies in *AP1*-*LFY.* Functional redundancies were detected in *AP1*-*CAL*, *AP2-AP1* and *AP2-LFY* genes [56,57]. *AP1* and *CAL* belong to the MADS domain genes and *UFO* codes for an F-box protein which degrades other proteins through a ubiquitation pathway [46,58,59]. *LFY* is also thought to promote *CAL* and *UFO* expression [60,61]. Therefore, these FMI genes cooperate together to promote the transition from vegetative growth to reproductive growth. Sequence homologs for *FMI* genes *CAL*, *AP2*, *LFY* and *AP1* were all identified in our *C. sinense* transcriptome database. These information of flowering integration genes and FMI genes would facilitate more detailed studies on the mechanism of floral differentiation for *C. sinense*.

### Differential expression of genes among three developmental phases

We have identified the putative homologs of *COL* and other key genes involved in controlling flowering time in *C. sinense*. More studies on their expression patterns and on their interactive relationships in the future could be used to elucidate the molecular mechanisms that regulate the floral transition and the flowering genetic pathway in *C. sinense*. To better understand the information related to gene expression obtained for *C. sinense*, we created three DEG libraries to analyze the gene expression patterns under three developmental phases. In the comparison between FDP with VP, most of the expressed genes were down-regulated and parts were up-regulated. In contrast, when RP was compared to VP and FDP, most of the expressed genes were up-regulated (Figure 7). Furthermore, a large amount of genes which showed specific expression in the three phases were likely involved in floral development.

When FDP was compared to VP, *TFL1* showed specific expression in VP. *TFL1* codes for a flowering repressor that can function to control inflorescence architecture and extend the phases of shoot meristems in *Arabidopsis*[40]. Our sequence was consistent with this theory. Among all specific expression of annotated genes in RP compared to VP or FDP, auxin response factors (ARF2) were identified in RP. Auxin signaling is important in the regulation of developmental transitions such as seed germination, induction of flowering, leaf senescence and shedding of senescent organs. ARFs are transcription factors that mediate responses to the plant hormone auxin [62]. For example, ARF2 promotes the transition between multiple stages of *Arabidopsis* development, including initiation of flowering, rosette leaf senescence, floral organ abscission and silique ripening. In contrast, ARF1 is a transcriptional repressor and the *arf1* mutation increases the transcription of Aux/IAA genes in *Arabidopsis* flowers [63]. Moreover, most of the specific gene expression was not annotated as a relative function, which could assist in the search for some new genes associated with floral development.

Although the molecular functions of *C. sinense* genes and the associated floral genetic pathways remain unknown, the present transcriptome analysis provides valuable information regarding *C. sinense* development, which could facilitate further investigations of the detailed floral development mechanisms of this culturally important orchid*.*

## Conclusion

The combination of RNA-seq and DGE analysis based on Illumina sequencing technology provided comprehensive information on gene expression. Candidate genes on flowering time genes, *MADS-box* genes and *COL* genes were rapidly identified by this approach. This data could be used as a tool to investigate the flowering pathway and various other biological pathways in *C. sinense.*

## Methods

### Plant materials and growth conditions

**Figure 10:**
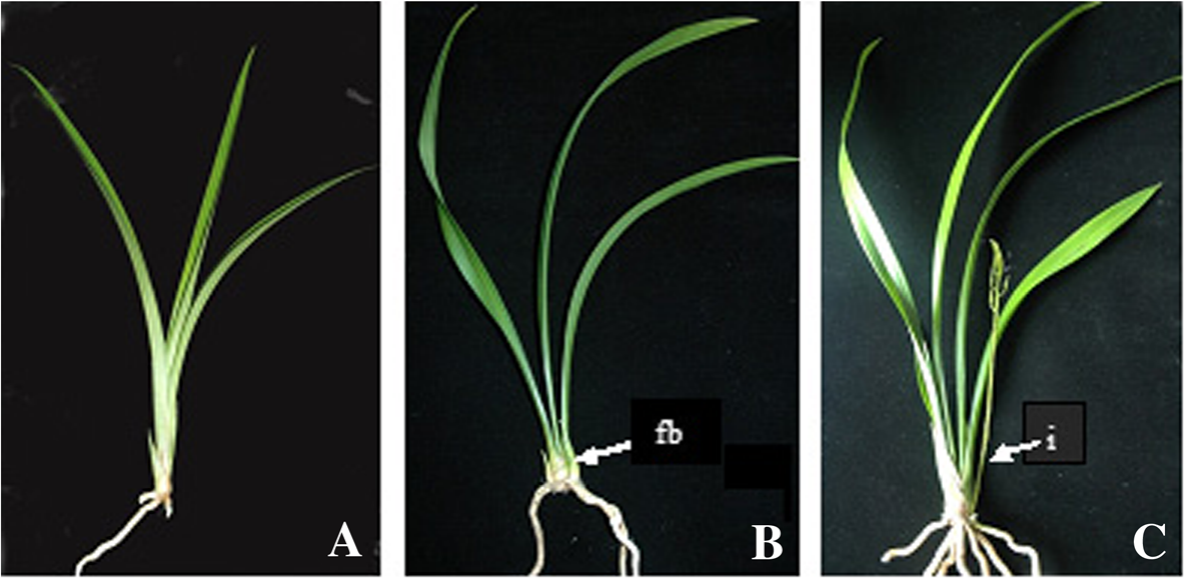
**Organs from *C. sinense *used to prepare DGE libraries for Illumine sequencing.** (**A**) A young *C. sinense* plant in the vegetative phase (VP); (**B**) A mature *C. sinense* plant with floral bud (fb) in the floral differentiation phase (FDP); (**C**) A mature *C. sinense* plant with inflorescence (i) in the reproductive phase (RP).

*C. sinense* ‘Qi Jian Bai Mo’ plants used in this study were grown and maintained in pots in a greenhouse of the South China Botanical Garden at a day/night temperature of 28/25°C with a 12-h period. The cDNA libraries were prepared from the entire plant of *C. sinense* at vegetative and reproductive stages. The vegetative phase (VP) samples were collected from a non-pseudobulb shoot (Figure 10A). The floral differentiation phase (FDP) samples were collected from the entire adult plant which had developed floral buds at the base of the pseudobulb (Figure 10B). The reproductive phase (RP) samples were collected from entire adult plants which had developed a peduncle with floral organs at the base of the pseudobulb (Figure 10C).

Fresh samples were used to extract total RNA immediately.

### cDNA library preparation and Illumina sequencing for transcriptome analysis

Total RNA was extracted using Column Plant RNAout2.0 (Tiandz, Inc. Beijing, China) according to the manufacturer’s protocol. To obtain complete gene expression information, a pooled RNA sample including roots, leaves, pseudobulbs, flower buds, young and mature inflorescences was used for transcriptome sequencing analysis. According to the Illumina manufacturer’s instructions, poly(A)^+^ RNA was purified from 20 mg of pooled total RNA using oligo(dT) magnetic beads. Fragmentation buffer was added to interrupt mRNA to short fragments. Using these short fragments as templates, a random hexamer-primer was used to synthesize the first-strand cDNA. Second-strand cDNA was synthesized using 10×buffer, 25 mM dNTPs, 20-60 U/μl RNaseH and 5 U/μl DNA polymerase I. Short fragments were purified with the QiaQuick PCR extraction kit and resolved with EB buffer for end reparation and for adding poly(A). Thereafter, the short fragments were connected with sequencing adapters. After agarose gel electrophoresis, suitable fragments were selected for PCR amplification as templates. Finally, the library was sequenced using Illumina HiSeq™ 2000. All raw transcriptome data were deposited in the GeneBank Short Read Archive (Accession SRA058042).

### Analysis of Illumina sequencing results

Raw reads from the image data output from the sequencing machine were generated by base calling. After filtering raw reads, *de novo* assembly of the transcriptome was carried out with a short reads assembling program – Trinity [64]. Trinity connects the contigs and obtains sequences defined as unigenes.

The generated unigenes were used for BLASTX and annotation against protein databases, including non-redundant (nr), SwissPort, COG, KEGG, and GO protein database, with a cut-off E-value of 0.00001. GO (http://www.geneontology.org) has three ontologies: molecular function, cellular component and biological process. With nr annotation, we used the Blast2GO program [65] to obtain the GO annotation of unigenes. After obtaining the GO annotation for every unigene, we used WEGO software [66] to perform GO functional classification for all unigenes and to understand the distribution of gene functions. KEGG is a major public pathway-related database [67] that is able to analyze a gene product during a metabolic process and related gene function in cellular processes. With the help of the KEGG database, we can further study genes’ biological complex behaviors, and by KEGG annotation we can obtain pathway annotation for unigenes. The KEGG pathways annotation was performed using Blastall software against the KEGG database.

### Digital gene expression (DGE) library preparation and sequencing

Total RNA was extracted from VP, FDP and RP using Column Plant RNAout2.0. mRNA was enriched by using oligo(dT) magnetic beads. After adding the fragmentation buffer, the mRNA was interrupted to short fragments (about 200 bp). Then the first strand cDNA was synthesized by a random hexamer-primer using the mRNA fragments as templates. 10×buffer, 25 mM dNTPs, 20-60 U/μl RNaseH and 5 U/μl DNA polymerase I were added to synthesize the second strand. The double-stranded cDNA was purified with a QiaQuick PCR extraction kit and washed with EB buffer for end repair and single nucleotide A (adenine) addition. Finally, sequencing adaptors were ligated to the fragments. The required fragments were purified by agarose gel electrophoresis and enriched by PCR amplification. The library products were ready for sequencing analysis via Illumina HiSeq™ 2000. Three raw DGE data (VP, FDP and RP) were deposited in the GeneBank Short Read Archive (accession SRA058974, SRA058977, SRA058978, respectively).

### Analysis and mapping of DGE tags

To map the DGE tags, the sequenced raw data were filtered to remove low quality tags (tags with an unknown nucleotide “N”), empty tags (no tag sequence between the adaptors) and tags with only one copy number (which might result from sequencing errors). For the annotation of tags, clean tags containing CATG and 21-bp tag sequences were mapped to our transcriptome reference database using SOAPaligner/soap2 [68]. Mismatches of no more than two bases were allowed in the alignment.

### Screening of differentially expressed genes (DEGs)

To compare the differences in gene expression at different developmental stages, the tag frequency in the different DGE libraries was statistically analyzed according to the method described by Audic and Claverie [14]. The false discovery rate (FDR) was used to determine the threshold P-value in multiple tests. We used FDR < 0.001 and an absolute value of the log_2_ ratio >1 as the threshold to determine the significant difference in gene expression. The differentially expressed genes were used for GO and KEGG enrichment analyses according to a method similar to that described by Xue [69]. GO terms, which take the corrected P-value ≤ 0.05 as a threshold, are significantly enriched in DEGs. KEGG pathways with a Q-value ≤ 0.05 are significantly enriched in DEGs.

Genes with similar expression patterns usually mean functional correlation. We perform cluster analysis of gene expression patterns with cluster [70] software and Java Treeview [71] software. In Figure 8, each column represents an experimental sample (e.g. VP, FDP and RP), each row represents a gene. Expression differences are shown in different colors. Red means high expression and green means low expression.

### Quantitative real-time PCR (qRT-PCR) validation

Total RNA was extracted as described for the DGE library preparation and sequencing. Each RNA sample was treated with RNase-free DNase (Promega) following the manufacturer’s protocol in an effort to remove any residual genomic DNA (gDNA). DNase-treated RNA (2 mg) was subjected to reverse transcriptase reactions using oligo-dT primer and PrimeScript™ Reverse Transcriptase (Takara) according to the manufacturer’s protocol.

The sequences of the specific primer sets are listed in Additional file 11. The actin gene of *C. sinense* (ACC NO. GU181353) was used as an internal gene. qRT-PCR was performed using the SYBR Premix Ex Taq Kit (TaKaRa) according to the manufacturer’s protocol. The results were normalized to the expression level of the constitutive actin gene. A relative quantitative method (DDCt) was used to evaluate the quantitative variation.

## Abbreviations

DGE: Digital gene expression; GO: Gene ontology; KEGG: Kyoto encyclopedia of genes and genomes; AP1: Apetala1; AP2: Apetala2; AP3: Apetala3; AG: Agamous; CAL: Cauloflower; CCA1: Circadian clock associated 1; CHS: Chalcone synthase; CO: Constans; COL: Constans-like; DEF: Deficiens; ELF3: Early flowering 3; EST: Expressed sequence tag; EMF2: Embryonic flower 2; FD: Flowering locus D; FE: Flowering locus E; FT: Flowering locus T; FLC: Flowering locus C; FWA: Flowering wageningen; GI: Gigantea; GLO: Globosa; LHY: Late elongated hypocotyl; LFY: Leafy; PDF2: Protodermal factor2; PIF3: Phytochrome interacting factor 3; PI: Pistillata; SEP: Sepallata; SOC1: Suppressor of overexpression of co 1; TFL1: Terminal flower 1; TOC1: Timing of cab expression 1; VIN3: Vernalization insensitive 3; VRN1: Vernalization 1; UFO: Unusual floral organs.

## Competing interests

The authors declare that they have no competing interest.

## Authors’ contributions

JZ carried out the experiments, performed the bioinformatics analyses, and drafted the manuscript. KW cultured and provided the experimental material. SZ participated in the qRT-PCR experiment. JATdS critically evaluated the protocol, the data and interpretation and revised the manuscript. HX performed the bioinformatics analyses. JD designed the study and revised the manuscript. XZ and CT participated in the design and coordination. All authors read and approved the final manuscript.

## Supplementary Material

Additional file 1 Metabolic pathway analysis result for unigenes by KEGG annotation. Click here for file

Additional file 2 **The unigenes that share homology with *MADS-box *genes.** Click here for file

Additional file 3 **The unigenes that share homology to *CONSTANS*-like genes.** Click here for file

Additional file 4 Specific expression of genes in the comparison between FDP with VP. Click here for file

Additional file 5 Gene set enrichment analysis in the comparison between FDP with VP by GO and KEGG. Click here for file

Additional file 6 Specific expression of genes in the comparison between RP and VP. Click here for file

Additional file 7 Gene set enrichment analysis in the comparison between RP with VP by GO and KEGG. Click here for file

Additional file 8 Specific expression of genes in the comparison between RP and FDP. Click here for file

Additional file 9 Gene set enrichment analysis in the comparison between RP and FDP by GO and KEGG. Click here for file

Additional file 10 **The functional annotation for the unigenes related with flower development in *C. sinense *DGE.** Click here for file

Additional file 11 Primer sequences for qRT-PCR. Click here for file
